# Thermo-Electro-Mechanical Characterization of PDMS-Based Dielectric Elastomer Actuators

**DOI:** 10.3390/ma15010221

**Published:** 2021-12-28

**Authors:** Konrad Katzer, Anas Kanan, Sascha Pfeil, Henriette Grellmann, Gerald Gerlach, Michael Kaliske, Chokri Cherif, Martina Zimmermann

**Affiliations:** 1 Institute for Material Science, Faculty of Mechanical Science and Engineering, Technische Universität Dresden, 01062 Dresden, Germany; Martina.Zimmermann@tu-dresden.de; 2 Fraunhofer Institute of Material and Beam Technology IWS, 01277 Dresden, Germany; 3 Institute for Structural Analysis, Faculty of Civil Engineering, Technische Universität Dresden, 01062 Dresden, Germany; michael.kaliske@tu-dresden.de; 4 Institute of Solid State Electronics, Faculty of Electrical and Computer Engineering, Technische Universität Dresden, 01062 Dresden, Germany; Gerald.Gerlach@tu-dresden.de; 5 Institute of Textile Machinery and High Performance Material Technology, Faculty of Mechanical Science and Engineering, Technische Universität Dresden, 01062 Dresden, Germany; Chokri.Cherif@tu-dresden.de

**Keywords:** dielectric elastomer actuators, mechanical hysteresis, thermal behavior, silicone, screen printing, solid state electrode, voltage dependencies, FEM simulation

## Abstract

The present contribution aims towards a thermo-electro-mechanical characterization of dielectric elastomer actuators (DEA) based on polydimethylsiloxane (PDMS). To this end, an experimental setup is proposed in order to evaluate the PDMS-based DEA behavior under the influence of various rates of mechanical loading, different ambient temperatures, and varying values of an applied electric voltage. To obtain mechanical, electro-mechanical and thermo-mechanical experimental data, the passive behavior of the material, as well as the material’s response when electrically activated, was tested. The influence of the solid electrode on the dielectric layer’s surface was also examined. Moreover, this work focuses on the production of such DEA, the experimental setup and the interpretation and evaluation of the obtained mechanical hysteresis loops. Finite element modeling approaches were used in order to model the passive and the electro-mechanically active response of the material. A comparison between experimental and simulation results was performed.

## 1. Introduction

**Figure 1:**
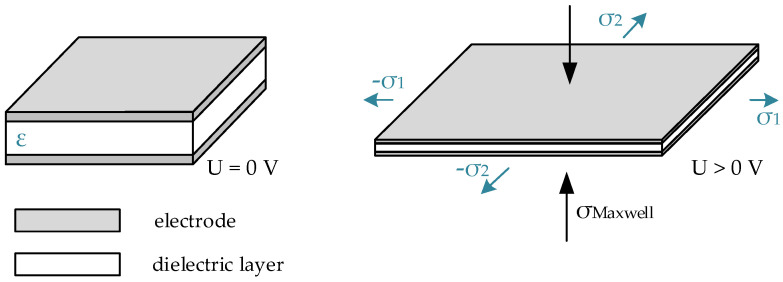
Schematic representation of the deforming mechanism of a dielectric elastomer actuator caused by Maxwell pressure.

Dielectric elastomer actuators are considered electroactive polymers. This means that if an electrical voltage is applied, they undergo a certain response, usually a deformation process. Since deformation strains bigger than 100% were reported by Pelrin et al. [1], the number of publications increased rapidly. DEA are used in a wide variety of areas but find their most frequent use in soft robotics and sensor application [2,3,4,5,6,7,8]. They are lightweight, their laboratory-scale production is reasonable and their deformation potential is outstanding [9]. The deformation behavior of dielectric elastomer actuators is caused by Maxwell stresses that are generated due to an applied electrical field. An electrical field can be induced in an orthogonal direction to the material’s surface by applying an electric potential difference between two conducting electrodes that cover the material’s surfaces. This leads to an equibiaxial, planar actuation of the structure [10]. In Figure 1, the electro-mechanical actuation mechanism is shown on a simple DEA, where $\sigma_{Maxwell}$ is the Maxwell stress, and $\sigma_{1}$ or $\sigma_{2}$ is described as the axial stress, which leads to an expansion of the dielectric layer. In order to calculate the electrostatic pressure $p_{el}$, an approach from Pelrine et al. [11] was used where $\varepsilon_{r}$ is the relative permittivity of the dielectric layer (the dielectric constant) and $\varepsilon_{0}$ is the permittivity of the free space (vacuum). The other variables are the electric field strength E, the Voltage U and the layer thickness of the dielectric layer t. In Equation (1), the connection between an applied electric field and the corresponding Maxwell stresses is shown. With increasing voltages, the electrostatic pressure increases quadratically:

(1) $$p_{el}=\varepsilon_{r}\varepsilon_{0}E^{2}=\varepsilon_{r}\varepsilon_{0}{\left(\frac{U}{t}\right)}^{2}.$$

Different types of DEA are developed based on acrylic VHB^TM^, hydrogels or polydimethylsiloxane (PDMS) [12,13,14]. While the majority of characterization studies can be found for acrylic VHB^TM^-based DEA, because of their uncomplicated handling and wide availability, PDMS-based materials offer many advantages. They are highly flexible, thin and unaffected by most chemicals. Additionally, in most cases, they offer a significantly lower viscoelastic behavior in comparison to acrylic VHB^TM^ sheets. In case scenarios that require fast activation and accurate movements, an almost purely elastic material is preferable. PDMS-based materials offer a soft and tactile surface and are therefore well-suited for applications in the field of gripping or handling fragile objects. Especially for soft robotics, those actuators offer great possibilities to achieve complex movements [2,5].

VHB^TM^-based materials were tested in varying test scenarios concerning the acquisition of material parameters influenced by the electric field. These findings are used as a validation basis for finite-element-method (FEM) simulation [15,16,17,18,19]. The research previously conducted showed a great effect on the viscoelasticity of these acrylic VHB^TM^ materials during loading–unloading cycles. Mehnert et al. showed that even after several minutes, there is still observable stress relaxation in VHB^TM^ [18]. The parameter identification for a suitable constitutive model was also conducted by Mehnert et al. [18], but, as stated before, only for VHB^TM^ materials.

In addition to the dielectric layer, the electrodes of a DEA have a crucial influence on the actuation behavior. In most cases, the electrodes of DEA are neglected while calculating the behavior of the active material. In contrast, Carpi et al. [20] showed that the electrodes indeed influence the behavior of the actuator. The mechanical behavior of the electrodes is difficult to predict and control. Therefore, the influence of the electrode layers should be as low as possible to ensure the most extensive process control over the DEA during activation. In order to achieve this, the electrode materials should be thin in comparison to the dielectric layer. From a mechanical behavior viewpoint, the electrodes should maintain a low Young’s modulus and should have properties as similar as possible to the dielectric material [21]. Electrodes pastes, such as carbon grease, have nearly no influence on the dielectric layer while being difficult to handle and showing low electrode quality. It is also possible to apply graphite powder without any carrier on the dielectric layer.

The greatest drawback with these kinds of electrodes is that they have poor structural integrity [20]. Due to the poor adhesion of these electrodes, solid but elastic electrodes are mostly used for the manufacturing of DEA. Elastomer-based electrodes that adhere to the dielectric layer are, in most cases, two-component liquid systems, e.g., silicone with introduced electrical conductive fillers. The main goal is to introduce as few conductive particles as possible inside an elastomeric matrix while keeping a low electrical resistivity. Mostly carbon-based fillers such as carbon nanotubes (CNT) or carbon black (CB) are used for this purpose. In the case of two-component PDMS-elastomers, it is possible to introduce the filler into the resin part before adding the hardening agent.

Additionally, the application method used in order to provide the dielectric layer with electrodes on both sides is of great importance. Different methods such as airbrushing, pad printing, inkjet printing or screen printing are evaluated in [22,23,24]. Screen printing offers great advantages for electrode patterns and usability. Whether the effects of screen printing parameters have an influence on the actuation behavior was investigated by Fasolt et al. [25]. It could be shown that any such effect is so minimal that it is negligible.

In order to evaluate the material’s behavior and the influence of different surrounding conditions, uniaxial test scenarios were widely used to characterize both the tensile and the compressive behavior of the material [18,21,26].

This publication focuses both on the fabrication of PDMS-based DEA and its mechanical characterization. Material parameters of the dielectric layer were compared to properties of the DEA. Thermal and electrical effects were investigated with respect to their effect on the DEA. Stress–strain relations are evaluated by plotting mechanical hysteresis loops. Finally, results from FEM simulation are compared with experimental results. This characterization approach was also used by various authors in order to evaluate the mechanical and electrical behavior [14,17,18,19,26]. The possibility to measure the mechanical, electrical and thermal properties in one description is the main advantage of the proposed characterization.

## 2. Materials and Methods

### 2.1. Production Process of DEA

The production process applied in this study is explained in the following. The electrode material was prepared in a two-stage process. An amount of 1.5 wt. % of single-walled CNT NC7000 (Nanocyl SA., Sambreville, Belgium) was added to the resin of Sylgard 184 (Dow Silicones, Wiesbaden, Germany). A premix was prepared inside a dual asymmetric centrifuge DAC 150 (Hauschild GmbH and Co. KG, Hamm, Germany). The predispersed resin with CNT was then processed inside a three-roll-mill EXAKT 80E (EXAKT GmbH, Norderstedt, Germany) with adjustable roll-gaps. A four-stage dispersion was performed with gap sizes starting from 90 µm down to 15 µm. The received resin was then mixed with the hardener in a 1:10 weight ratio and again mixed inside a dual asymmetric centrifuge.

An Elastosil 2030 (Wacker Chemie AG, Munich, Germany) silicone film with a thickness of 200 µm was used as a dielectric layer. The compliant electrodes were applied directly onto the dielectric layer using the screen printing method. This method relies on a mesh to transfer a certain geometry with ink on a substrate. The mesh had a yarn count of 43 cm^−1^. The screen printing machine Flat DX-100 (Siebdruckversand, Magdeburg, Germany) was used in order to apply the opposite electrodes congruently. The liquid electrode paste was flooded inside the screen with a squeegee with a Shore A hardness of 80. Thermal curing was performed at 50 °C for 24 h to ensure a complete solidification of the electrode ink.

**Figure 2:**
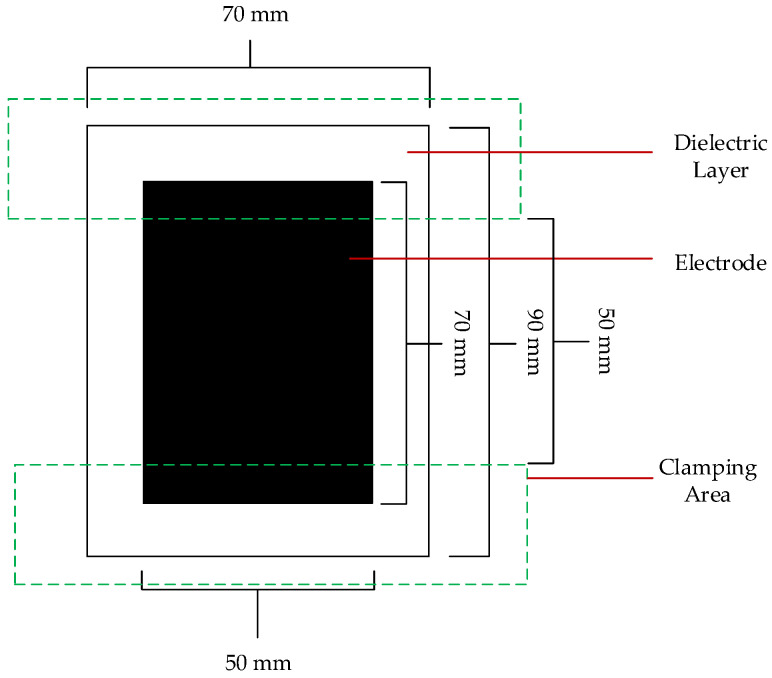
Layout of the finished DEA with corresponding sizes and marked clamping areas.

The size of the finished DEA can be seen in Figure 2. The clamping area is also marked, and it visualizes where the electrodes are in contact with the conducting part of the clamping arrangementThese tailored clamping arrangements were applied to both sides of the DEA in order to ensure adequate functionality of the DEA. The clamping device with the DEA was then slid into the tensile test machine. Overall, the free size of the DEA inside the tensile test machine is 50 mm in height and 70 mm in width, and the free, not clamped electrode is 50 mm × 50 mm in dimensions.

### 2.2. Test Setup for Thermo-Electro-Mechanical Measurements

In order to compare the influences of the intrinsic and external influence parameters, different test scenarios were carried out. First, the dielectric layer was examined without an electrode and compared to the behavior of an unactivated DEA with electrodes. Mechanical hysteresis loops were recorded from the DEA at voltages ranging from 0 V to 5000 V in steps of 1000 V. Additionally, the influence of different surrounding temperatures (−10 °C; 23 °C and 40 °C) were tested in a climate chamber Mytron KPK 70 (mytron Bio und Solartechnik GmbH; Heilbad Heiligenstadt, Germany). The mechanical hysteresis loops were evaluated in a tensile test machine Zwick 050 (Zwick and Roell GmbH; Ulm, Germany) at two different strain rates, 0.1 s^−1^ and 0.02 s^−1^, with a 100 N force transducer Z6FD1 (HBM, Darmstadt, Germany) with a maximum strain of 50%. Three hysteresis loops were recorded for each test. For a detailed analysis of the mechanical behavior, one of the hysteresis loops applied was analyzed in detail.

**Figure 3:**
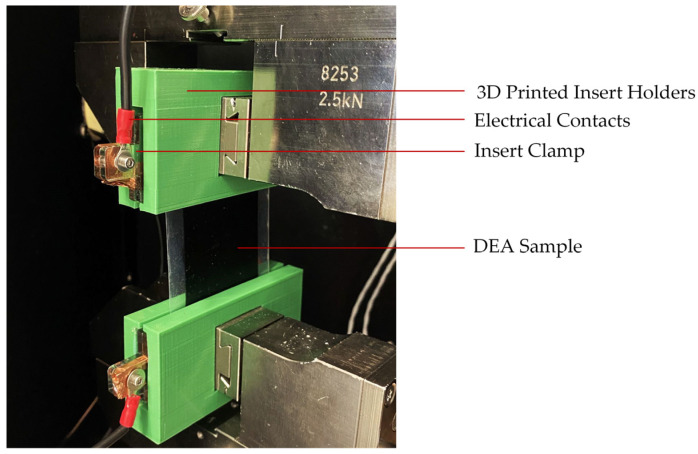
Test setup inside the temperature chamber with 3D-printed insert clamp holder and DEA sample.

In order to ensure a proper clamping of the DEA inside the tensile test bench, a custom-made clamping jaw was designed. The test setup is shown in Figure 3. In order to reduce safety risks, the DEA and the electrical contacts had to be isolated against the machine using only non-conducting materials to separate the metal clamps and the sample.

**Figure 4:**
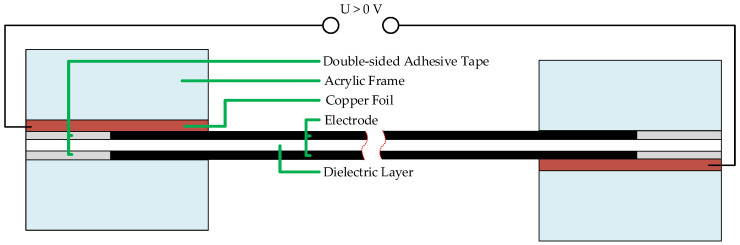
Schematic representation of the inserts for the insert holders with a DEA sample shows the contacting of the compliant electrodes.

Another important step in clamping the DEA samples was to ensure that clamping pressure applied to the electrodes should be kept as low as possible. Therefore, as visualized in Figure 4, a special holding device was designed. The adhesion of the DEA to the clamps was achieved using 3M, 91022 (3M, Neuss, Germany) double-sided adhesive tape. The insert with the adhered DEA sample was placed loosely inside the 3D-printed insert holder and carefully tightened with the metallic clamps.

### 2.3. Finite Element Modelling Approach

The passive behavior of the material is considered viscoelastic, where the mechanical properties are electric-field-dependent. To this end, an electro-viscoelastic material model and the corresponding finite element framework at large deformations were used to simulate the mechanical and electro-mechanical experiment [27]. A total energy density function $\Psi^{tot}$ is decomposed into a volumetric part of the equilibrium response $\Psi_{e}^{tot}$, an isochoric part of the equilibrium response $\Psi_{e}^{iso}$ with an electric field dependency of material parameters, an isochoric part of the non-equilibrium response $\Psi_{v}^{iso}$ and a coupled contribution $\Psi^{coup}$ as

(2) $$\Psi^{tot\;}\left(J,\bar{C},{\bar{C}}^{e},E\right)=\Psi_{e}^{vol}\left(J\right)+\Psi_{e}^{iso}\left(\bar{C},E\right)+\Psi_{v}^{iso}\left({\bar{C}}^{e}\right)+\Psi^{coup}\left(\bar{C},E\right)\;.$$

In Equation (2), J=det (F) is the Jacobian of the deformation gradient, $\bar{C}$ is the total volume-preserving part of the right Cauchy–Green deformation tensor, ${\bar{C}}^{e}$ is the elastic volume-preserving part of the right Cauchy–Green deformation tensor and E is the electric field in the reference configuration.

The volumetric response of the material is described by

(3) $$\Psi_{e}^{vol}\left(J\right)=\frac{\kappa }{4}\left(J^{2}-2\ln J-1\right).$$

In Equation (3), κ is the bulk modulus of the material. The isochoric hyperelastic response of the material is specified by the extended tube model [28], which reads

(4) $$\Psi_{e}^{iso}\left(\bar{C},E\right)=W_{e}\left({\bar{I}}_{\bar{C}},E\right)+L_{e}\left({\bar{\lambda }}_{a},E\right),\;W_{e}\left({\bar{I}}_{\bar{C}},E\right)=\frac{G_{c}\left(E\right)}{2}\left[\frac{\left(1-\delta^{2}\right)\left({\bar{I}}_{\bar{C}}-3\right)}{1-\delta^{2}\left({\bar{I}}_{\bar{C}}-3\right)}+\ln\left(1-\delta^{2}\left({\bar{I}}_{\bar{C}}-3\right)\right)\right],\;L_{e}(\lambda^{-},E)=\frac{2G_{e}(E)}{\beta^{2}}\sum_{a=1}^{3}({\bar{\lambda }}_{a}^{-\beta }-1)$$

In Equation (4), $\Psi_{e}^{iso}$ is split into a cross-linking part $W_{e}$ and a topological tube $L_{e}$ with the parameter δ that expresses limited chain extensibility and the parameter β that describes topological constraints. Moreover, ${\bar{I}}_{\bar{C}}=\mathrm{tr}\bar{C}$ denotes the first invariant of the isochoric Cauchy–Green tensor and {${\bar{\lambda }}_{a}=1\;to\;3$} are the isochoric principal stretches. The material parameters $G_{C}\left(E\right)$ and $G_{e}\left(E\right)$ contribute to the total shear modulus of the material and are assumed to be electric field dependent in the form

(5) $$G_{c}\left(E\right)=G_{c0}+{\hat{G}}_{c}E\cdot E,\;G_{e}\left(E\right)=G_{e0}+{\hat{G}}_{e}E\cdot E.$$

In Equation (5), $G_{c0}$ and $G_{e0}$ are ground state contributions. Moreover, ${\hat{G}}_{c}$ and ${\hat{G}}_{e}$ are material parameters that express the dependency of $G_{e}\left(E\right)$ and $G_{c}\left(E\right)$ on the electric field E, respectively. A similar approach to the one shown in Equation (5) was used in [29] to express the dependency of mechanical parameters on the magnetic field and in [18] to describe the dependency of mechanical parameters on the electric field. For the description of the non-equilibrium behavior, a Neo-Hookean energy expression is specified in terms of the first invariant of the elastic part of the isochoric right Cauchy–Green tensor ${\bar{I}}_{{\bar{C}}^{e}}$ as

(6) $$\psi_{v}^{iso}\left({\bar{c}}^{e}\right)=\frac{1}{2}G_{v}\left({\bar{I}}_{\bar{c}}^{e}-3\right).$$

In Equation (6), the parameter $G_{v}$ is the shear modulus related to the non-equilibrium response. The evolution of the elastic part of the deformation can be connected to the rate of inelastic deformation ${\tilde{d}}_{v}$. Referring to the approach of Bergström and Boyce [30], the tensor ${\tilde{d}}_{v}$ can be introduced as

(7) $${\tilde{d}}_{v}=\dot{\gamma }N_{p}.$$

In Equation (7), $\dot{\gamma }$ denotes the effective rate of creep and should be greater than zero ($\dot{\gamma }>0)$. The normalized projection tensor $N_{p}$ can be introduced as

(8) $$N_{P}=\frac{\tau_{V}^{iso}}{||\tau_{v}^{iso}|{|}^{\prime }},\;\;\mathrm{where}\;\;||\tau_{v}^{iso}||=\sqrt{\tau_{v}^{iso}:\tau_{v}^{iso}}.$$

In Equation (8), $\tau_{v}^{iso}$ is the viscous Kirchhoff stress. An evolution law for the effective rate of creep rate as suggested in [1] is adopted. The evolution law takes the form

(9) $$\dot{\gamma }={\dot{\gamma }}_{0}\lambda_{p}^{c}{\left(\frac{\tau_{v}}{\tau }\right)}^{m}\;\mathrm{with}\;\tau_{v}=\frac{\left||\tau_{v}^{iso}|\right|}{\sqrt{2}},\;{\bar{\lambda }}_{e}=\sqrt{\frac{{\bar{I}}_{{\bar{c}}^{e}}}{3}}.$$

The evolution law, as shown in Equation (9), depends on $\tau_{v}$ and the elastic stretch ${\bar{\lambda }}_{e}$. Furthermore, the material parameters in Equation (9) are restricted to $\frac{{\dot{\gamma }}_{0}}{\tau^{m}}>0,c\geq 0$ and m>0.

The electro-mechanically coupled contribution $\Psi^{coup}$ is specified as

(10) $$\Psi^{coup}\left(\bar{C},E\right)=c_{1}E\cdot E+c_{2}E\cdot \bar{C}\cdot E$$

In Equation (10), the parameter $c_{1}$ affects the electrical polarization only, and the parameter $c_{2}$ influences both electrical polarization and electro-mechanical coupling of the material [31]. An analytical solution for electro-elasticity with the coupling behavior considered as shown in Equation (10) is detailed in [32]. A similar description of the coupled contribution as shown in Equation (10) was previously used in combination with a description similar to the one given in Equation (5) to simulate electro-mechanical experiments, where both an electrical field and a mechanical loading are applied simultaneously [18]. Through presenting several simulation–experimental investigations, it was shown by [18] that the use of Equations (5) and (10) are adequate to model the associated electro-mechanical experiments at large strain. Regarding the numerical solution, a mixed Q1P0 electro-mechanical finite element formulation was utilized [27].

## 3. Results

The following part is separated according to the influence factors due to the different test scenarios. First, the effect of the electrode on the basic dielectric layer was tested. Subsequently, the influence of temperature and voltage in the active state was evaluated. The last stage was to correlate the simulation results with the experimental data. The results presented are intended as a fundamental study on the mechanical behavior of the DEA processed. Therefore, the actual data discussed are presenting tendencies; the reproducibility and likely scatter in the values were not in the focus of this study.

### 3.1. Effect of the Electrodes on the Dielectric Layer

**Figure 5:**
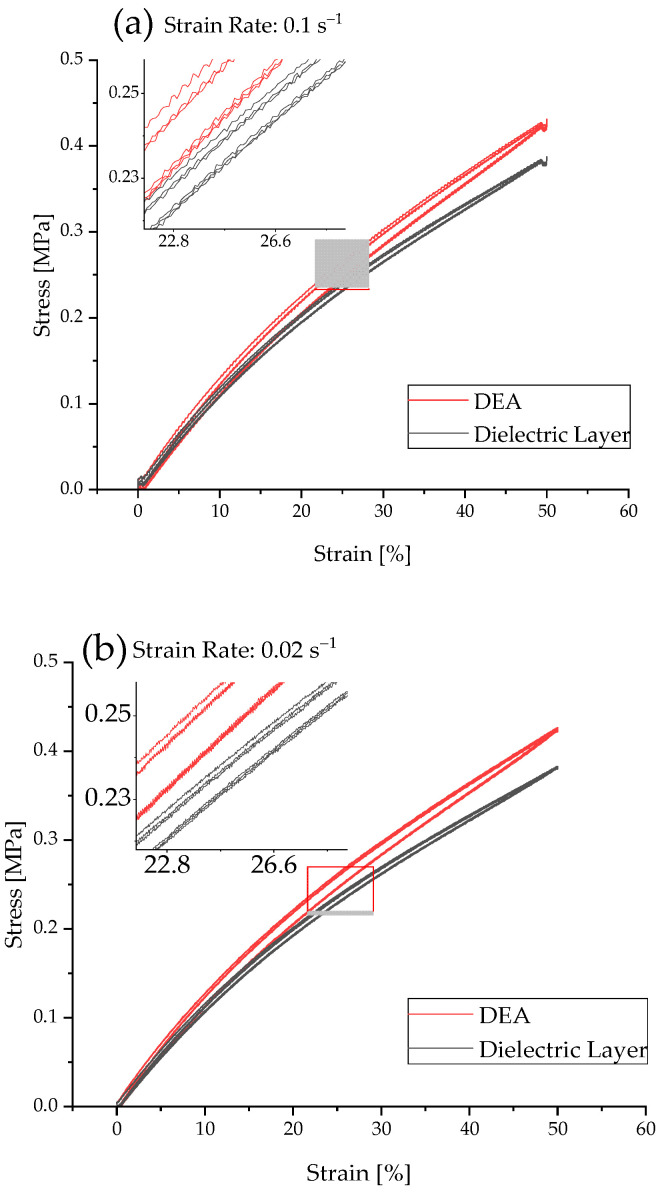
Stress–strain hysteresis at a strain rate of (**a**) 0.1 s^−1^ and (**b**) 0.02 s^−1^ of a DEA and of the pure dielectric layer.

In order to evaluate the effect of the printed electrode on the dielectric layer, tests were performed using 200 µm thick dielectric membranes with and without surface-coating electrodes. The hysteresis curves represent one sample each and are shown in Figure 5.

The effect of a printed electrode is clearly visible for both strain rates. Especially in the range above 15% of strain, the strain stiffening in the DEA becomes visible. The maximum stress of the dielectric layer amounts to 0.388 MPa (0.02 s^−1^ strain rate) and 0.383 MPa (0.1 s^−1^ strain rate). The DEA shows an increase in the maximum stress to 0.427 MPa (0.02 s^−1^ strain rate) and 0.431 MPa (0.1 s^−1^ strain rate), respectively. The higher strain rate leads to an overall increase in the maximum stress values. This is due to the entropic effect of the material, which leads to higher stresses at higher strain rates caused by a hindered entanglement of the polymeric chains. The amount of hysteresis in the DEA is high compared to the amount of hysteresis in the dielectric layer without an electrode. This increase in the quantity of hysteresis could be caused by the filler inside the electrode material. The filler may hinder the elastic PDMS network in a way that leads to flaws which enhances the viscoelastic effects and softens the matrix. Additionally, it was visible that with the increasing strain rate, the noise of the stress–strain curves increased. This is due to the vibration of the tensile testing machine.

### 3.2. Temperature Dependencies

**Figure 6:**
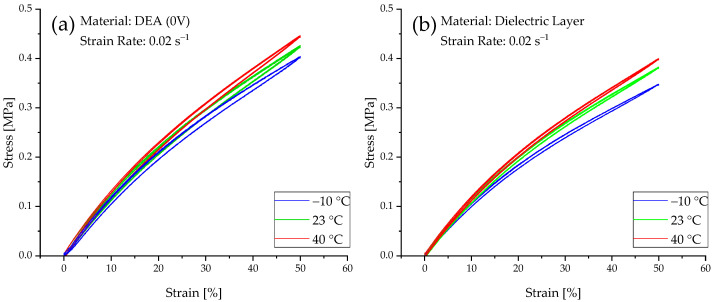
Stress–strain hysteresis at a strain rate of 0.02 s^−1^ for the (**a**) DEA (no activation) and (**b**) dielectric layer at different temperatures.

The influence of varying temperatures on the dielectric layer alone and on the DEA without an applied voltage can be seen in Figure 6. The experiments with a strain rate of 0.02 s^−1^ are shown. The experiments at a higher strain rate depict a similar influence. Both graphs show that with decreasing temperature, the curve flattens. The maximum stress for the DEA decreases from 0.446 MPa at 40 °C via 0.427 MPa at 23 °C to 0.404 MPa at −10 °C. The same progression can be seen for the dielectric layer but with lower maximum stress values, starting from 0.400 MPa at 40 °C via 0.383 MPa at 23 °C to 0.348 MPa at −10 °C. This leads to a reduction in stress values. Moreover, the flattening of the hysteresis loop is due to the entropic behavior of the elastomeric network. At higher temperatures, the chains are more likely to be hindered from entanglement. Therefore, larger stresses are induced in the material. At lower temperatures, the molecular movement is decreased, and as a result of this, the chains can align themselves more easily along the load path. Additionally, the entanglement in the entropic elastic range, which is commonly under 100% strain for PDMS-elastomers, is an exothermic process that benefits from lower temperatures. Furthermore, a slightly lower amount of hysteresis is seen for the purely dielectric layer in comparison to the DEA. Normally, when charging the DEA, because of the applied current and the relatively high resistance of the electrodes, the DEA heats up. Since the DEA are only charged before the experiment, this effect can be neglected here.

### 3.3. Effect of Voltage and Temperature on the Active Behavior

**Figure 7:**
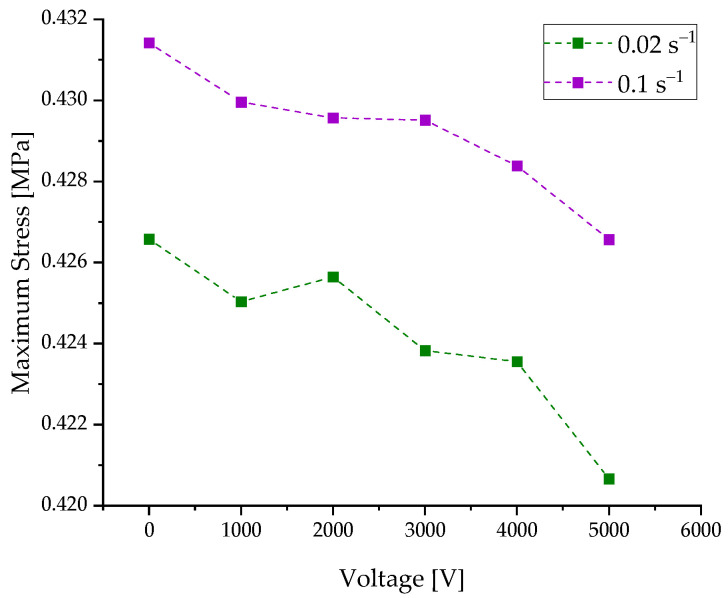
Voltage dependencies of the maximum stress values during the hysteresis loops for two different strain rates (0.02 s^−1^ and 0.1 s^−1^).

In order to show the effect of varying applied voltage on the DEA, the voltage is correlated with the maximum stress at each voltage level at 23 °C, as shown in Figure 7.

The maximum stress decreases at 23 °C with increasing voltage for a strain rate of 0.1 s^−1^ from 0.431 MPa at 0 V to 0.426 MPa at 5000 V, and for a strain rate of 0.02 s^−1^, it is from 0.426 MPa at 0 V to 0.420 MPa at 5000 V. As expected, the maximum stress for the lower strain rate is below that of the higher strain rate. The decrease in the maximum stress with increasing voltage is probably caused by force acting in the direction of the load cell along the load path due to the electrostatic pressure $p_{el}$. This leads to a reduction in the total forces and, as a consequence, on the mechanical stresses. Additionally, the overall stress level is higher with higher strain rates. The same explanation as in Figure 5 applies. A higher strain rate leads to a hindered entanglement of the polymeric chains.

**Figure 8:**
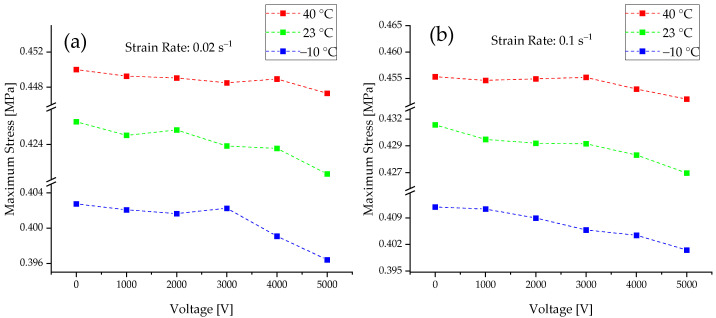
Voltage and temperature dependencies of the maximum stress values during the hysteresis for (**a**) strain rate 0.02 s^−1^ and (**b**) 0.1 s^−1^.

**Figure 9:**
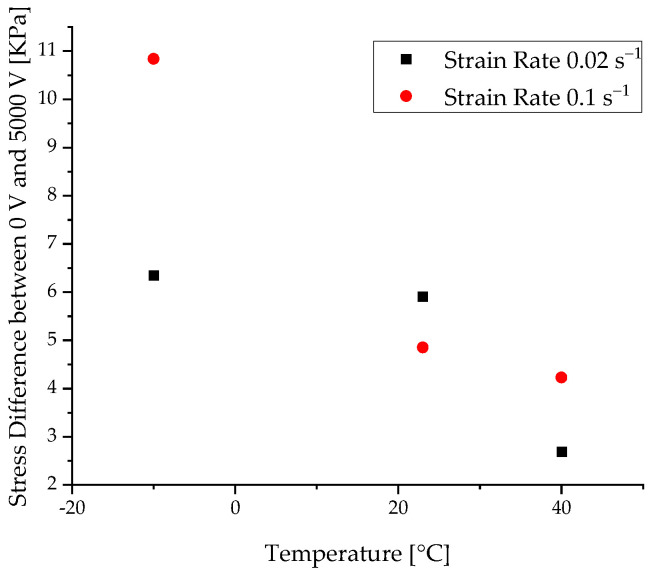
Voltage and temperature dependencies of the difference between maximum stress at 0 V and 5000 V.

As already expected from unactuated DEA results, the same effect can be observed for the actuated DEA. With a falling temperature, the maximum stress level also decreases, as seen in Figure 8. It is considered that this behavior is caused by the entropic process of the chain entanglement, which is exothermic and therefore is preferentially effective at lower temperatures. If the differences between the unactivated state at 0 V of a DEA are compared to the 5000 V level, and the differences are plotted against the temperature, it is clearly visible that, with increasing temperature, the produced force of the actuator decreases significantly. This shows that the actuation amount of the DEA depends on the temperature and that the working point of a DEA should be at rather low temperatures. This correlation is shown in Figure 9. This effect is present for both strain rates. Since the stretching of the elastomer is an exothermic process and thus hinders itself, the stretching capacity is increased by low temperatures.

### 3.4. Correlation with Simulation Results

**Figure 10:**
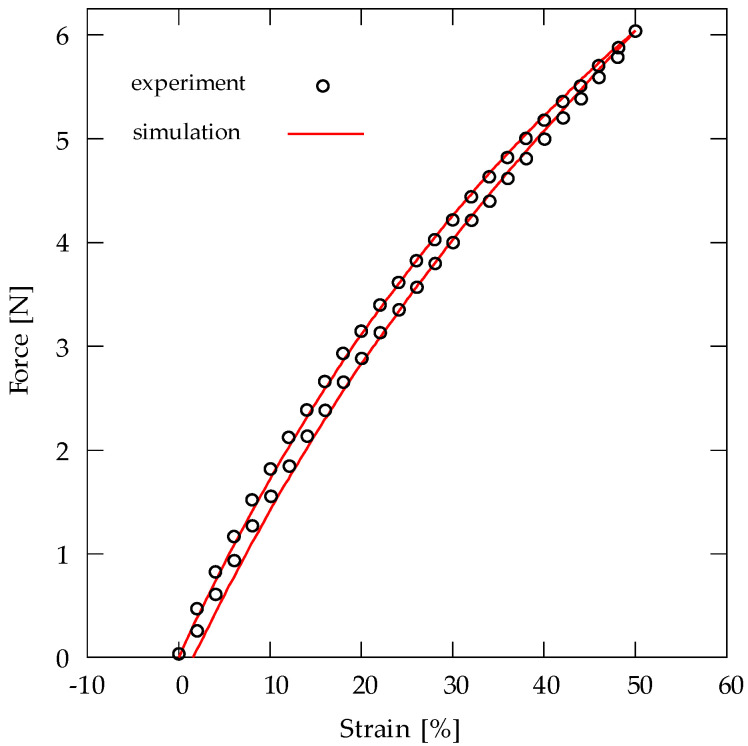
Experimental data and simulation results for strain–force relation of the loading–unloading test with a strain rate $\dot{\epsilon }=0.1\;s^{-1}$.

**Figure 11:**
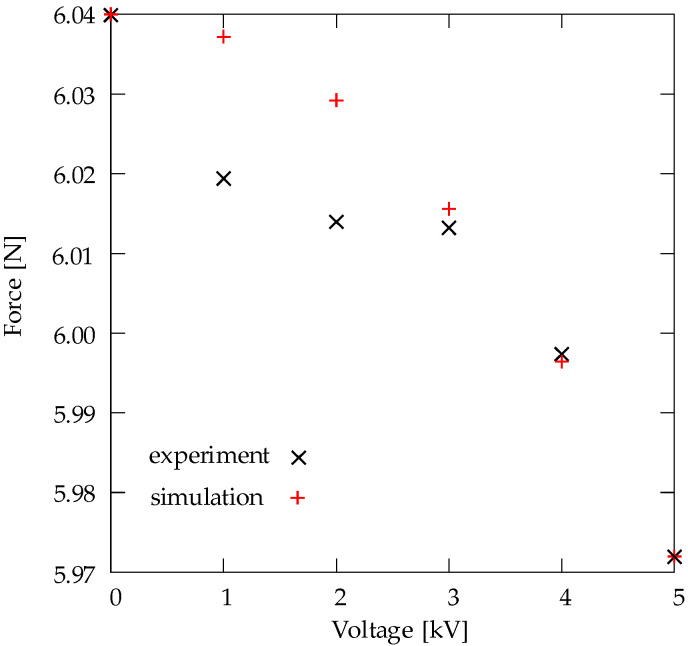
Experimental data and simulation results of the relation between an applied voltage and the maximum force.

The electro mechanical behavior of a specimen with dimensions 70 × 50 × 0.2 mm³ was considered and analyzed. The thickness of the surface-coating electrodes is assumed to be negligible, which is an assumption used for modeling dielectric elastomer actuators in several contributions, for example, see [17,18]. A passive loading–unloading test was simulated, where one end of the specimen was fixed, and the other end was driven with a strain rate $\dot{\epsilon }=0.1\;s^{-1}.$ It is sought to fit experimental results by choosing suitable material parameters. The hyperelastic and viscous material parameters used for the simulation of the passive test are shown in Table 1. The simulation and the experimental results of the passive test are depicted in Figure 10. Regarding the active electro-mechanical response, simulations of several loading–unloading tests are performed by applying various constant potential differences Δϕ = {0, 1, 2, 3, 4 and 5} kV, across the thickness of the loaded specimen. The applied mechanical boundary conditions are similar to the boundary conditions used for the passive test. The passive material parameters of the model are chosen based on fitting the simulation and the experimental results for the passive loading–unloading test. The electrical and electro mechanical parameters are identified by fitting the simulation and the experimental results for Δϕ = 5 kV. The parameters used for the simulation are shown in Table 1. By using the identified material parameters, the electro-mechanical response is simulated for Δϕ = {1, 2, 3 and 4} kV. Figure 11 depicts simulation and experimental results of the relation between the applied voltage and the maximum resulting force. The experimental results align relatively well with the simulation results for Δϕ = {3 and 4} kV, as shown in Figure 11. However, for Δϕ = {1 and 2} kV, it can be noted that there is a relatively large difference between the experimental and the simulation predictions, as depicted in Figure 11.

Regarding the active electro-mechanical response, simulations of several loading–unloading tests are performed by applying various constant potential differences Δϕ = {0, 1, 2, 3, 4 and 5} kV, across the thickness of the loaded specimen. Figure 11 depicts simulation and experimental results of the relation between the applied voltage and the maximum resulting force. The passive material parameters of the model are chosen based on fitting the simulation and the experimental results for Δϕ = 0 kV. The electrical and electro-mechanical parameters are identified by fitting the simulation and the experimental results for Δϕ = 5 kV. By using the identified material parameters, the electro-mechanical response is simulated for Δϕ = {1, 2, 3 and 4} kV.

Figure 11 shows that the experimental results align relatively well with the simulation results for Δϕ = {3 and 4} kV. However, for Δϕ = {1 and 2} kV, it can be noted that there is a relatively large difference between the experimental and the simulation predictions.

## 4. Summarizing Discussion

At first, several tests were performed in order to show the effect of the electrode on the dielectric layer. The results are similar to the experimental findings of Kleo et al. [33], who concluded that the existence of electrodes on the dielectric layer leads to an increase in stiffness. Additionally, the results show the effect of different temperatures on the voltage-dependent, active behavior of DEA as well as strain rate dependencies of maximum stress values and mechanical hysteresis loops. Increasing temperature leads to a raised stress level. This is mostly explained by exothermic processes during the occurrence of chain entanglement. With increasing voltage, the maximum stress at the end of the loading cycle decreases. This is caused by the electrostatic pressure that builds up between the activated electrode and the equibiaxial force, which also acts in the direction of the load path. This lowers the amount of force needed to elongate the material until a fixed elongation is reached. The correlation between simulation and experimental data shows a good overall alignment of the data. With some exceptions, the modeling approach for the FEM simulation is suitable. Minor discrepancies will need to be examined in more detail in the future but are probably a result of deviations of the experimental results due to the test setup and imperfections in the DEA. One of the main limitations of the proposed characterization is the uniaxial test setup. In order to measure the equibiaxial behavior, a different characterization method needs to be used, where the force path has to be introduced in all planar directions equally.

An elongation of 50% strain is relatively low compared to other publications [17,18] but can be explained by a rather high default rate and electric breakdown of the DEA at higher strains. The experiments were also repeated for higher elongations of up to 120%. However, the default rate was too high in order to continue the experiments. Only lower elongations resulted in an acceptable failure rate. In future experiments, the film thickness of the dielectric layer should be decreased, which would lead to a higher electro-mechanical coupling, but at the same time, increase the risk of an electrical breakdown. It is also expected that, with higher maximum strain and decreased dielectric layer thickness, the simulation results fit the experimental data better because the effect of the electro-mechanical coupling becomes bigger. Therefore, the deviations caused by the test setup can become smaller, and especially in areas with low voltage, the methodical error influence decreases. Future research should additionally focus on extending the thermal ranges. Temperatures above 40 °C and below −10 °C should show the effect seen in Figure 6 even more prominently.

## Figures and Tables

**Table 1:** Material parameters.

Parameter Type	Value
Hyperelastic material parameters	κ=170.0 MPa$,\;G_{C0}=0.2353\;\mathrm{MPa}$, $G_{e0}=0.102358\;\mathrm{MPa}$, δ=0.0 [−], β=1.0 [−].
Viscous material parameters	$G_{v}=0.1\;\mathrm{MPa}$$,\;\frac{{\dot{\gamma }}_{0}}{\tau^{m}}=0.8\;{\mathrm{MPa}}^{-m}s^{-1}$, c=0.0 [−], m=1.4 [−].
Electro-mechanical parameters	$c_{1}=-2.65626\;\times \;10^{-11}\;N/V²$ $c_{2}=1,32813\;\times \;10^{-11}\;N/V²$ ${\hat{G}}_{c}=-1.13\;\times \;10^{-12}\;N/\left(\mathrm{Vmm}\right)²$ ${\hat{G}}_{e}=-3.42549\;\times \;10^{-13}\;N/\left(\mathrm{Vmm}\right)²$

## Data Availability

All the data is available within the manuscript.
